# The YARHG Domain: An Extracellular Domain in Search of a Function

**DOI:** 10.1371/journal.pone.0035575

**Published:** 2012-05-17

**Authors:** Penny Coggill, Alex Bateman

**Affiliations:** Wellcome Trust Sanger Institute, Wellcome Trust Genome Campus, Hinxton, United Kingdom; Ludwig-Maximilians University, Germany

## Abstract

We have identified a new bacterial protein domain that we hypothesise binds to peptidoglycan. This domain is called the YARHG domain after the most highly conserved sequence-segment. The domain is found in the extracellular space and is likely to be composed of four alpha-helices. The domain is found associated with protein kinase domains, suggesting it is associated with signalling in some bacteria. The domain is also found associated with three different families of peptidases. The large number of different domains that are found associated with YARHG suggests that it is a useful functional module that nature has recombined multiple times.

## Introduction

Protein domains are important building blocks of proteins. They often act as the common currency of protein function that nature rearranges to create novel activities [1]. Identification of novel protein domains can identify proteins that are related both in terms of their function and their evolutionary history. Despite the long history of domain discovery [2], we appear still to be a long way from having a comprehensive set of all the functional domains in nature. In this article we identify a small novel extracellular protein domain that, due to its co-occurrence in proteins with other well-studied domains, we hypothesise acts to bind peptidoglycan or some other as yet unidentified ligand.

During the construction of the Pfam database of protein domains [3] we are trying to identify regions of conservation that might represent new domains. One approach that we have been using recently is to take all the proteins from a bacterial proteome that are not already matched by Pfam, and begin iterative searches with them. In many cases these searches find proteins that belong to well-known families that have not so far been picked up by the existing Pfam profile-HMMs (Hidden Markov Models). However, there have also been a large number of apparently new families identified using this method. In a recent screen of the bacterium *Fusobacterium nucleatum sub spp vincentii* [4] we identified a new domain in the protein expressed from gene FNV1478 that we have termed the YARHG domain after a set of conserved residues found within many of its related sequences.

## Methods

The YARHG domain was initially identified using the Jackhmmer program, which is part of the HMMER3 package written by Sean Eddy [5]. The Jackhmmer program is similar in terms of function to the PSI-BLAST program [6]. Jackhmmer takes an initial sequence and builds a profile-HMM from it and searches a protein database. All of the homologues which are more significant than the inclusion threshold (default of 0.001) are included in a new multiple sequence-alignment. This alignment is then used to build a new profile-HMM that is again searched against the sequence database. This cycle continues until either the profile-HMM finds no new homologues or the maximum number of iterations is reached (default 5 rounds). In our hands Jackhmmer appears to be more sensitive than PSI-BLAST, but it is a little slower to run. This is supported by the work of Johnson *et* *al*. who published a preliminary version of Jackhmmer [7].

Predictions of the secondary structure of the domains were carried out using the Jpred3 software [8], and all proteins were run through Phobius to predict signal-peptides and trans-membrane regions [9].

To aid in discovery of the function we attempted to identify other domains that are associated on the same sequence with the YARHG domain. To identify associated domains we searched all the proteins found in the family against the Pfam database.

A phylogenetic tree was generated from a representative sequence-alignment using Phymol 3.0 with default parameters: http://www.atgc-montpellier.fr/phyml/, to generate a bootstrapped (100) phylip tree in Newick-format. For the same sequences a corresponding phylip-interleaved file of all the domain architectures was produced manually. These two files were run together through the iTOL web-browser at: http://itol.embl.de/upload.cgi, to produce a phylogenetic tree with associated domain-architectures.

## Results and Discussion

**Figure 1:**
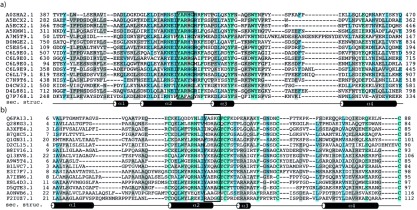
Multiple sequence-alignments of the YARHG and YASKG domains. (a) Multiple sequence-alignment of the full YARHG domain created using the MUSCLE alignment tool [34] and coloured using the belvu software's default colouring scheme to highlight conserved residues [35]. The eponymous YARHG motif is enclosed in a black box. Secondary structure elements shown have been predicted with the Jpred3 server (http://www.compbio.dundee.ac.uk/www-jpred/) using the YARHG domain sequence-region (residues 311 to 396) of A5KMW1 as the query-sequence. (b) Multiple sequence-alignment of the YASKG subgroup of the YARHG domain. A black box identifies the YARHG-equivalent region in the alignment. Likewise, secondary structure elements are shown derived from the sequence-region (residues 6 to 88) of Q6FA13 as the query-sequence.

We initiated the Jackhmmer search with the uncharacterised protein-product of gene FNV1478 (UniProt Knowledgebase accession: Q7P768.1), using default parameters, against the UniProtKB database [10]. This search identified 295 distinct sequence-regions (on 290 distinct proteins) sharing conservation with the C-terminus of the FNV1478 protein, all from bacteria both Gram-positive and Gram-negative. A multiple sequence-alignment of this region is shown in Figure 1a. The YARHG domain has been deposited in Pfam and assigned the accession number PF13308, and is available in Pfam release 26.0. The N-terminal part of the FNV1478 protein from residues 1 to 220 was identified as a second conserved domain that we have called DUF4424 (for Domain of Unknown Function 4424) that has been assigned accession number PF14415. A further 24 sequences also carry this DUF associated with the YARHG domain.

The conserved region of the YARHG domain is 70 amino acids in length and shows a number of conserved residues that are likely to be important for its structure and function. In addition to the YARHG motif there is a highly conserved C-terminal glutamate residue as well as two pairs of conserved aromatic amino acid residues. Although the conserved charged arginine (from the YARHG motif) and the glutamate might be suggestive of an enzymatic domain, it is unusual for such short domains to carry out any enzymatic activity. The secondary-structure predictions identify a set of four alpha-helices (see Figure 1), suggesting that the domain might form a four-helix bundle.

The YARHG domain is an extracellular domain. In almost every case it is found in a protein that possesses a signal-peptide, targeting the protein for secretion, and/or contains one or more trans-membrane helices, with the YARHG domain predicted to be extracellular. One of the few exceptions to this is in the original query protein FNV1478. Alignment of this protein to its close relatives shows that it is truncated at the N-terminus relative to its homologues that possess a predicted signal peptide. We thus suggest that the initiating methionine of the FNV1478 protein-product has been incorrectly predicted.

One subgroup of YARGH domain proteins contains the motif YASKG in place of the YARGH motif as shown in Figure 1b. These proteins share four completely conserved cysteine residues that are likely to form two disulfide bridges. An iterative jackhammer search against the database with one of these sequences, the protein from *Acinetobacter sp*. strain ADP1 (UniProtKB accession: Q6FA13_ACIAD), finds only other YASKG- and YARHG-carrying sequences. Although these proteins are quite distinctive in sequence from the classical YARGH domain members their predicted secondary-structure is comparable (see Figure 1b)

**Figure 2:**
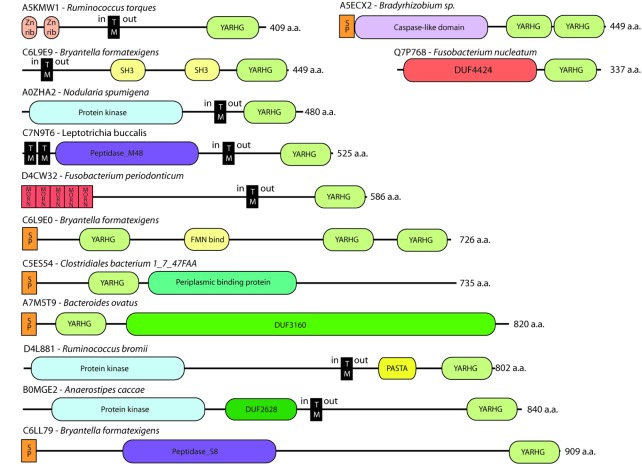
Representative domain-architectures of proteins containing the YARHG domain. One representative sequence-graphic for each of the 14 different combinations of domains that the YARHG domain is found with. Note that the formal name for *Bryantella formatexigens* is now *Marvinbryantia formatexigens* [25].

To aid in discovery of the function we have attempted to identify any other domains that are associated on the same sequence with the YARHG domain. We have found a surprisingly large number of different domain-organisations represented, suggesting that this domain has an important function that has been re-used several times during evolution. Protein domains that are fused together onto the same sequence often share a common function [11], so, by looking at the nature of its associated domains, we may find clues as to the function of the YARHG domain. To identify associated domains we searched all the proteins found in the family against the Pfam database. We identified 14 different domain-architectures, as shown in Figure 2.

There are many proteins where the YARHG domain is found in isolation, or at least with no other known domains. These YARHG-only proteins form the majority (174 out of a total of 290) of identified proteins. One feature of the YARHG domain is that it is very frequently found at the C-terminus of proteins, and most of such proteins have a signal-peptide at their N-terminus.

### Zinc-ribbon

The YARHG domain is frequently found (in 73 instances) associated with a pair of N-terminal zinc-ribbon domains (Pfam accessions: PF12773, PF13240, PF13248). Zinc-ribbon domains are often involved in nucleic acid-binding [12]. The protein domain-architecture for the example in Figure 2, A5KMW1, also contains a central trans-membrane helix, suggesting that the zinc-ribbon is intracellular and the YARHG domain is extracellular; this organisation is found to be common to all the sequences with these zinc-ribbons. We hypothesise that protein A5KMW1 is a membrane-tethered transcription factor [13], with the extracellular YARHG domain binding to some as yet unidentified extracellular ligand that causes a signal to be passed into the cell leading to a transcriptional response. There are several known examples where bacterial transcription factors are sequestered in the membrane until they are released by an external signal [13], and a particularly good example of where *E. coli* uses an extracellular domain to sense changes in the external pH, signalling this through the membrane, is the case of CadC. CadC, a membrane-integrated transcriptional activator, is released only at low pH when it then binds directly to the DNA in the cadBA operon and alters gene expression [14].

### Protein Kinases

There are six sequences where the YARHG domain is associated with a protein serine/threonine kinase (Pkinase) domain (Pfam accession: PF00069). Four of these proteins come from Gram-negative Cyanobacteria, see example A0ZHA2. These four sequences are all very similar. They are approximately 500 residues long with no signal-peptide; however, they all have a trans-membrane helix between the N-terminal Pkinase and the C-terminal YARHG domain, suggesting that the YARHG domain is extracellular. None of them has any other associated domains.

The remaining two sequences carrying a Pkinase domain are from Gram-positive Firmicutes, see D4L881 and B0MGE2 in Figure 2. In each of these proteins the Pkinase domain is found at the N-terminus of the protein, with the YARHG domain at the C-terminus separated by a trans-membrane alpha helix. In B0MGE2 there is an associated DUF2628 domain of unknown function (Pfam accession: PF10947), and in D4L881 a PASTA domain (Pfam accession: PF03793) is found adjacent to the YARGH domain.

In all of these six cases the architectural arrangement hints at some form of cross-membrane signalling, whereby the YARHG might be detecting some external ligand whose binding triggers the Pkinase to phosphorylate downstream (intracellular) protein substrates. The PASTA domain binds to beta-lactam antibiotics and is suggested to bind to unlinked peptidoglycan cross-links [15], suggesting that the YARHG domain might also be involved in peptidoglycan binding.

The YARHG domain is, additionally, found in a single protein (E1L326) associated with a SPOR domain (Pfam accession: PF05036). This domain has also been shown to bind to peptidoglycan.

### Peptidases

Three sequences from different species, from different bacterial clades (Proteobacteria, Fusobacteria and Firmicutes), carry an associated peptidase domain, although the peptidase-type is different in each case. The UniProtKB sequence accession A5ECX2 from *Bradyrhizobium sp* contains a Caspase-like cysteine-aspartic protease (Pfam accession: PF00656) with a pair of tandem repeated YARHG domains at the C-terminus. This protein has a signal-peptide so is secreted. This is the only sequence from this nitrogen-fixing *Bradyrhizobium* genus to carry a YARHG domain [16]. Caspases are enzymes that initiate and execute the cascade of cellular disassembly referred to as programmed cell death [17]. Programmed cell death was thought to be confined to protists, plants and fungi, but the discovery of caspase homologues in many bacterial and Archaeal species – see Pfam family accession: PF00656 and [18], [19] – often referred to as metacaspases, suggests that these may represent an initial, ancestral, core of executioners that have led to the emergence of the cell death machinery [20].

Metallopeptidases of the MEROPS M48 family [21] (Pfam accession: PF01435) in bacteria are induced by heat-shock and may be involved in the degradation of abnormal or unstable membrane proteins [22]. The *E. coli* Htpx protein has three trans-membrane regions whereas our M48 example, C7N9T6, has five, suggesting that it might be behaving slightly differently. Gram-negative bacterial proteases may be involved in regulated intra-membrane proteolysis (RIP) [23], where RIP proteases are polytopic integral membrane proteins that cleave their substrates within the membrane. In Figure 2, the architecture for C7N9T6 might represent one such RIP, as this protein does carry the motif GxxxN/SG (as GxxxS) downstream of the HExxH motif, a feature characteristic of these membrane RIP proteins [24].

Peptidase family S8, also known as the subtilisin family (Pfam accession: PF00082), is the second largest family of serine peptidases (MEROPS). Members of family S8 have a catalytic triad in the order Asp, His and Ser in the sequence, and have a characteristic GTSMA sequence-motif also found in the Firmicute sequence from *Marvinbryantia formatexigens*, C6LL79, example in Figure 2 [25]. This protein has no transmembrane helix although it is apparently targeted to the membrane as it has a signal-peptide at the N-terminus. Prokaryotic subtilisins are involved in a range of diverse functions [26] including the nutrition of the organism and its invasive activity, so this protein might be being targeted to the membrane to be cleaved and then passed out of the cell to digest a food-source substrate.

### Periplasmic-binding domain

Two Clostridial sequences each carry a long Periplasmic-binding protein superfamily domain [27] (Pfam accession: PF12849) just downstream of the YARHG domain. The Periplasmic-binding domains are involved in binding solutes and delivering them for uptake to membrane-transport systems. These proteins contain a signal-peptide targeting them for secretion.

### SH3

Two sequences from *Marvinbryantia formatexigens* carry a pair of SH3 domains (Pfam accession: PF08239). This bacterium is a Clostridial gut acetogen with the ability to consume oligosaccharides and boost the yield of succinate [28]. Again, these proteins have a single trans-membrane helix to the N-terminus of the SH3 and YARHG domains, which the PHOBIUS program predicts should lie outside the membrane. The SH3 domain is perhaps the best characterised of all the protein-interaction modules, but it was only discovered to occur in prokaryotes in 1999 [29]. Through its capacity to bind, with moderate affinity and selectivity, to proline-rich ligands, these domains are critical for a wide range of biological functions in eukaryotes. Their molecular functions in bacteria are not well understood, but they are widely found in proteins involved in cell wall biosynthesis, supporting the hypothesis that the YARHG domain might be binding to some component of the bacterial cell wall such as peptidoglycan [30].

### FMN-binding

One sequence from *Marvinbryantia formatexigens*, the Clostridial gut acetogen with the ability to consume oligosaccharides [28], carries an FMN-binding domain (Pfam accession: PF04205) associated with two tandem repeated YARHG domains, again with a signal-peptide. The FMN-binding domain interacts selectively and non-covalently with flavin mononucleotide, the prosthetic group of various oxidoreductases, serving as an electron carrier by being alternately oxidised (FMN) and reduced (FMNH2). There are only three proteins from this species that carry the FMN-binding domain and one of these is C6L9E0 – see Figure 2.

### MORN

Two sequences from *Fusobacterium spp* carry four MORN (membrane occupation and recognition nexus, Pfam accession: PF07661) repeats in tandem at their very N-termini, the YARHG domain in each one then lying extra-cellularly at the very C-terminus, separated from the MORNs by a single trans-membrane helix. These repeating sequence-motifs are found in junctophilins [31] and appear to be involved in linking the intracellular cytoskeleton [32] with the plasma membrane [33].

### Evolution of YARHG multi-domain architectures

**Figure 3:**
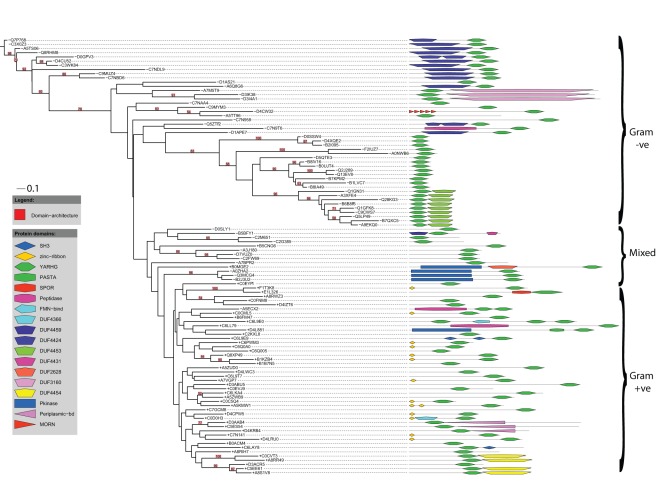
Phylogenetic distribution and domain-architecture of sequences carrying the YARHG domain. Phylogenetic tree of sequence-regions found in the YARHG family aligned with their relative domain-architectures. Bootstrap values >50 are indicated. The Gram-status of each sequence is shown by the relevant ‘+’ or ‘−’ in front of the accession number.

The species carrying the YARHG domain are distributed fairly evenly between Gram-positive and Gram-negative bacteria, with 50% being Firmicutes, the remainder being a mixture of Gram-negative Proteobacteria –20%– Fusobacteria –13% – Bacteroidetes –10%– and a handful of others including 3.5% Spirochaetes. The proteins that possess the exact YARHG sequence-motif are almost all Gram-positive, whereas the cysteine-containing YASKG subfamily member-species are all Gram-negative. To examine the phylogenetic distribution of the YARHG domain between the Gram-positive and Gram-negative bacterial species that carry it, we have produced a combined phylogeny and domain-architecture diagram as shown in Figure 3.

Phylogenetically, the Gram-positive and Gram-negative sequences are not clearly separated in the tree, but one should bear in mind that the analysis was run on just the short section of each sequence contained only in the YARHG domain. However, there are still large clades that contain only Gram-positive or Gram-negative sequences. By making multiple sequence-alignments of each type we noted that the Gram-positive YARGH domains conserve the YARGH sequence-motif much more strongly than the Gram-negative examples do.

The phylogeny of the YARGH domain proteins suggests that although many different domains are found associated on these sequences it is likely that most if not all domain-fusion events happened only on a single occasion in evolution. All sequences carrying the zinc-ribbons are on Gram-positive sequences, and their current distribution might suggest that all their more closely neighbouring sequences once carried such an N-terminal domain but that this has subsequently become lost over time along some lineages. The Peptidase domain is the only independent domain found on both Gram-positive and Gram-negative sequences, but in each of the three cases the peptidase is of an evolutionarily different class, a metallo-peptidase for C7N9T6, a cysteine-peptidase in A5ECX2 and a serine-peptidase for C6LL79. In general, any individual corresponding domain is associated with just one or at most two distinct clades, e.g., the group of Gram-negatives at the top of Figure 3 carrying DUF4424, and then the group of closely related Gram-positives carrying the Pkinase domain.

A question arises as to the mechanism of domain-gain in YARGH proteins. The two examples, of YARHG with DUF4453 among some Gram-negatives and of YARHG with DUF4454 among a small group of Gram-positives (see Figure 3), are interesting because these DUFs occur on virtually no other sequences, so on no other occasion without an associated YARHG domain. In the case of D3ACR5 that lacks a DUF4454 we found a sequence, D3ACR4, carrying just a DUF4454, indicating close proximity on the genome. Both these DUFs would appear to have been fused to the YARHG domain quite recently. So it seems likely that domain-accretion in the YARGH domain family has been due to loss of stop codons between previously adjacent but separate genes.

In this study we have identified a new protein domain that has been associated with a wide variety of other protein domains during evolution. Although the functional nature of this domain is uncertain we hypothesise that it binds to an as yet unknown ligand. We think that the most likely candidate for such a ligand is the peptidoglycan component of the bacterial cell wall. The domain is found in Gram-positive bacteria that have a thick peptidoglycan layer. In these organisms the domains strongly conserve the YARHG motif. In Gram-negative bacteria, which have a thinner peptidoglycan layer, the YARHG motif is much less well conserved suggesting that the domain might have either a more diverse range of ligands or that it no longer binds a ligand. Future functional and structural studies will be needed to elucidate in full the role that this domain plays.
